# Chemotherapy and the pediatric brain

**DOI:** 10.1186/s40348-018-0087-0

**Published:** 2018-11-06

**Authors:** Chrysanthy Ikonomidou

**Affiliations:** Department of Neurology, Section of Child Neurology, University of Wisconsin Madison, Madison, WI 53705 USA

**Keywords:** Neurotoxicity, Cognition, Brain injury, Disease mechanisms

## Abstract

Survival rates of children with cancer are steadily increasing. This urges our attention to neurocognitive and psychiatric outcomes, as these can markedly influence the quality of life of these children. Neurobehavioral morbidity in childhood cancer survivors affects diverse aspects of cognitive function, which can include attention, memory, processing speed, intellect, academic achievement, and emotional health. Reasons for neurobehavioral morbidity are multiple with one major contributor being chemotherapy-induced central nervous system (CNS) toxicity. Clinical studies investigating the effects of chemotherapy on the CNS in children with cancer have reported causative associations with the development of leukoencephalopathies as well as smaller regional grey and white matter volumes, which have been found to correlate with neurocognitive deficits.

Preclinical work has provided compelling evidence that chemotherapy drugs are potent neuro- and gliotoxins in vitro and in vivo and can cause brain injury via excitotoxic and apoptotic mechanisms. Furthermore, chemotherapy triggers DNA (deoxyribonucleic acid) damage directly or through increased oxidative stress. It can shorten telomeres and accelerate cell aging, cause cytokine deregulation, inhibit hippocampal neurogenesis, and reduce brain vascularization and blood flow. These mechanisms, when allowed to operate on the developing brain of a child, have high potential to not only cause brain injury, but also alter crucial developmental events, such as myelination, synaptogenesis, neurogenesis, cortical thinning, and formation of neuronal networks.

This short review summarizes key publications describing neurotoxicity of chemotherapy in pediatric cancers and potential underlying pathomechanisms.

## Introduction

The 5-year survival rate for childhood cancers exceeds 80%, resulting in a growing population of long-term survivors. One in 570 young adults between ages 20 and 34 years is a childhood cancer survivor [1], and 40% of them have at least one chronic medical condition which include neurocognitive toxicity [2–4]. In many cases, especially those from the late 1900s, neurocognitive toxicity is the result of combined polychemotherapy and radiation treatments. However, recent systematic multicenter longitudinal studies of intellectual development of childhood acute lymphoblastic leukemia (ALL) patients treated solely with polychemotherapy also document lower performance IQ (intelligence quotient) scores and worse intellectual outcomes in patients diagnosed and treated prior to the 6th year of life [5]. Given that the tumor burden reflected by low versus increased risk did not affect IQ scores, the investigators attributed this adverse effect to treatment rather than to the disease burden. In another study on pediatric B-cell ALL survivors, the authors described the development of leukoencephalopathies as late as 7.7 years after the end of treatment [6]. In this study, 40% of patients scored < 86 on either Verbal or Performance IQ. Children had significant attention problems and neurocognitive impairments, which were dependent upon treatment protocol. This strongly supports concerns about intensive chemotherapy being a major contributor to CNS late effects [6].

Deficits are consistently reported in visual processing, visual-motor function, attention, concentration, working memory, and executive functions [7–11]. Buizer and colleagues [9] reviewed 21 trials comparing patients with ALL and controls and described long-term deficits in attention and executive function, worse among the younger female patients. There are also studies which did not detect significant neurocognitive deficits in ALL survivors. Von der Weid and coinvestigators [12] found no significant differences between 132 survivors of ALL versus not-CNS solid tumors in global IQ. Jansen and colleagues reported in a prospective longitudinal, sibling-controlled study of children with ALL, treated with chemotherapy only, no major differences between patients and siblings up to 4.5 years from diagnosis except for a significant diminution in complex fine motor functioning in the patients at the last evaluation [13].

Understanding the pathomechanisms via which chemotherapy impacts on central nervous system (CNS) integrity is crucial to the development of cancer therapies that will spare the CNS.

Many studies have been performed in children with brain tumors and leukemia to explore chemotherapy impact on the developing CNS [14–16]. Studies of intellectual development of childhood acute lymphoblastic leukemia (ALL) patients treated solely with polychemotherapy document worse intellectual outcomes in patients diagnosed and treated prior to the 6th year of life [5]. Our understanding of how chemotherapy injures the pediatric brain, what the pathomechanisms of this injury are and what accounts for the higher vulnerability of children under 6 years of age remains limited. We know that chemotherapy associates with leukoencephalopathies and low white matter/grey matter volumes in pediatric B-cell ALL survivors, but we know very little about the biochemical and microstructural changes that lead to these states. More systematic research in this area is urgently needed in order to characterize mechanisms of chemotherapy neurotoxicity in children, identify biomarkers which signal critical CNS toxicity, and also design strategies to prevent it.

The great majority of clinical studies addressing neurotoxicity of chemotherapy in children with cancer are cross-sectional studies performed after cancer treatment has been completed. There are few studies focusing on dynamic changes in brain morphology and function and molecular changes in biological fluids during chemotherapy in children, and there is a dearth of longitudinal prospective clinical studies that examine timely progression and potential reversibility of evolving sequelae. This is the type of studies needed to help explore what acute effects cancer and cancer treatment exert on the developing brain, how early they occur, what the earliest indicators and mechanisms are, and whether treatments can be developed to counteract and/or prevent them.

### Effects of chemotherapy on the microstructure of the developing brain

The neural structures and circuits affected by chemotherapy treatment are beginning to be explored. Application of neuroimaging tools could help start to uncover a neural basis for the cognitive deficits observed in cancer survivors. With the advances and refinement of imaging technologies, it has become increasingly recognized that CNS-directed chemotherapy for ALL but also polychemotherapy for solid peripheral tumors lead to alterations in white (WM) and grey matter (GM) which are visible with modern imaging techniques.

#### White matter

A prospective longitudinal study assessing effects of chemotherapy on the WM in the pediatric brain reported on the occurrence of transient and mostly reversible WM changes during chemotherapy in the majority of patients [14]. Some cross-sectional studies provide evidence that chemotherapy alone or combined with radiation in children cause changes in the WM [15, 17–20]. Carey and colleagues [15] used voxel based morphometry (VBM) analysis in subjects who were treated with systemic and intrathecal chemotherapy only and reported reduced WM volumes in the right frontal lobes compared to healthy individuals. Others [18] used diffusion tensor imaging (DTI) analysis and examined the images of 13 adult survivors, 17–37 years old, who had been treated with total brain radiation and chemotherapy. These authors reported significantly reduced fractional anisotropy values in the temporal lobes, hippocampi and thalami, which were accompanied by significant WM volume loss. Reddick and coinvestigators [19] used voxel-based analysis of T2-weighted imaging of patients during treatment to identify which WM regions are preferentially damaged. Two sets of conventional T2-weighted axial images were acquired from 197 consecutive patients (85 female, 112 male; aged 1.0–18.9 years) enrolled on an institutional ALL treatment protocol. Two highly significant bilateral clusters of T2 signal intensity change were identified in both 1-group and 2-group analyses. Increased T2-weighted signal intensity from these regions both within and between examinations were nonlinear functions of age at examination, and the difference between the examinations was greater for older subjects who received more intense therapy. These analyses identified specific WM tracts involving predominantly the anterior, superior, and posterior corona radiata and superior longitudinal fasciculus, which were at increased risk for the development of T2-weighted hyperintensities during therapy for childhood ALL. The investigators concluded that these vulnerable regions may be the cause of subsequent cognitive difficulties consistently observed in survivors. Another group of investigators [21] aimed to determine if the loss of WM fractional anisotropy (FA), measured by DTI in post-treatment childhood medulloblastoma and acute lymphoblastic leukemia survivors, correlates with IQ scores. This was a cross-sectional study performed at 6.38 years after diagnosis of ALL and 3.25 years after diagnosis of medulloblastoma. Change in FA had a significant effect on full-scale IQ and verbal and performance IQ. It was suggested that WM FA may be a clinically useful biomarker for the assessment of treatment-related neurotoxicity in childhood cancer survivors [21].

A more recent study by Edelmann and colleagues [22] in survivors of childhood ALL treated with chemotherapy alone (*n* = 36), cranial radiation (*n* = 39), and healthy controls (*n* = 23) revealed that survivors of ALL treated with chemotherapy alone performed worse in processing speed, verbal selective reminding and academics compared to population norms. They also measured higher fractional anisotropy in fiber tracts within the left hemisphere and a lower ratio of WM to intracranial volume in frontal and temporal lobes. There were significant associations between neurocognitive performance and brain imaging, particularly for frontal and temporal WM and GM volumes. The predictive value of FA within the frontal lobe for neurotoxicity in childhood ALL survivors has been suggested [23]. Finally, atypical structural connectome organization in young survivors of ALL was described [24]. Clustered connectivity in the parietal, frontal, hippocampal, amygdalar, thalamic, and occipital regions was altered in the ALL group compared to control subjects and could underlie impaired local information processing, hub connectivity, and cognitive reserve [24].

Sleurs and colleagues [25] conducted magnetic resonance (MR) diffusion imaging in survivors of childhood bone and soft tissue sarcoma. This is the first study to show extensive regions with lower fractional anisotropy and fixel-based measures of apparent fiber density in survivors of solid peripheral tumors (non-ALL, non-CNS). The authors demonstrated global chemotherapy-related changes with particular vulnerability of centrally located WM bundles.

#### Grey matter

There are few studies examining GM changes during chemotherapy in cancer patients, mainly adults. McDonald and coinvestigators [26, 27] evaluated GM alterations in a cross-sectional MRI study in breast cancer patients with (*n* = 17) and without (*n* = 12) chemotherapy and in healthy controls (*n* = 18). The chemotherapy groups had decreased GM volumes in the bilateral frontal, temporal, and cerebellar regions and right thalamus at 1 month with some recovery seen at 1 year.

Genschaft and colleagues [16] performed a cross-sectional study of brain morphology and neurocognitive function in adolescent and young adult survivors of childhood ALL (*n* = 27), treated with chemotherapy only, and healthy controls (*n* = 27). Volumes of GM, WM, and olfactory bulbs were measured using FMRIB’s Integrated Registration and Segmentation Tool (FIRST) and voxel-based morphometry (VBM). The authors found smaller mean GM volumes of the left hippocampus, amygdala, thalamus, and nucleus accumbens in the ALL group. VBM analysis revealed significantly smaller volumes of the left calcarine gyrus, both lingual gyri and the left precuneus. Lower scores in hippocampus-dependent memory were measured in ALL subjects, while lower figural memory correlated with smaller hippocampal volumes. These findings demonstrate that childhood ALL treated with chemotherapy associates with smaller volumes of neocortical and subcortical GM and lower hippocampal memory performance in adolescence and adulthood [16].

Tamnes and colleagues [28] reported smaller surface area in several cortical regions including prefrontal regions, which associated with problems in executive functioning in childhood ALL survivors (ages 18–46 years; age at diagnosis 0–16 years; years since diagnosis 7–40). The pathomechanisms of these differences remain unclear, i.e., it is unknown whether the smaller GM volumes in the ALL groups are caused by destruction of neuronal tissue, impaired neuro- and gliogenesis or disturbance of structural refinements (cortical thinning) that occur naturally during development.

### Neurochemical biomarkers in body fluids in cancer patients

Few studies have focused on analysis of chemical and molecular biomarkers indicative of chemotherapy-induced CNS toxicity in cancer patients. Oesterlundh and colleagues [29] analyzed neurochemical markers of brain injury in cerebrospinal fluid (CSF) during induction treatment for acute ALL in children (*n* = 121; mean age 6.4 years/1.3–16.8 years) on days 0, 8, 15, and 29. They reported significant increases in the levels of neuron-specific-enolase, glial fibrillary acidic protein, and neurofilament protein light chain which suggest that cell injury, involving both neurons and astroglia, occurs during induction chemotherapy in children. Elevation of homocysteine and excitatory amino acid neurotransmitters in the CSF of children who receive methotrexate for cancer treatment has been reported [30] as well as increased beta-glucuronidase activity in the CSF of children with ALL undergoing treatment with high-dose methotrexate [31]. Beta glucuronidase levels correlated with plasma methotrexate levels. The authors concluded that increased beta-glucuronidase activity was due to enzyme leakage through the cell membranes caused by the toxic effect of methotrexate on the cells of the CNS [31].

In a small prospective study, it was shown that neuron-specific enolase CSF levels increase during induction chemotherapy for ALL and remain elevated during consolidation [32]. The authors also reported increased levels of nerve growth factor and brain-derived neurotrophic factor in the CSF during induction and consolidation therapy for ALL, which were interpreted as indicating activation of neuroprotective mechanisms.

Van Gool and colleagues [33] described increase in CSF-Tau, CSF-P-Tau, and CSF-neuromodulin after induction chemotherapy and one intrathecal injection of methotrexate followed by 7 days of systemic corticosteroids. CSF-Tau remained high during induction treatment whereas CSF-P-Tau and CSF-neuromodulin decreased suggesting different mechanisms of neurotoxicity in the course of induction chemotherapy. Similary, Krawzuk-Rybak and coinvestigators [34] measured elevated tau protein levels in the CSF of ALL patients. A negative correlation was found between Tau protein levels obtained from the last CSF (during last routine lumbar puncture) with total and verbal IQ, performance IQ, and perceptual organization index. They concluded that WM injury occurs during chemotherapy and that elevated Tau protein levels in the CSF at the end of treatment indicate future neurocognitive difficulties.

Higher degrees of oxidative stress in the CNS, as measured by levels of oxidized CSF phospholipids in 88 children undergoing chemotherapy for ALL, correlated with higher degree of cognitive dysfunction [35]. Elevated CSF levels of F2 isoprostanes (oxidative marker) and caspase 3/7 (apoptosis markers) were reported in three patients with methotrexate toxicity following intrathecal chemotherapy and high-dose methotrexate [36].

Finally, CSF folate and homocysteine levels were studied in patients with ALL [37]. CSF folate dropped during the first month of therapy and remained low throughout treatment. CSF homocysteine was inversely related to cognitive function prior to treatment and increased during treatment. Of 36 patients who had imaging after completion of chemotherapy, 9 had periventricular or subcortical white matter abnormalities consistent with leukoencephalopathy. In these patients, CSF peak tau concentrations were the highest suggesting that these biomarkers may have predictive value for neurologic outcomes in long-term survivors of childhood ALL [37].

### Mechanisms of chemotherapy-induced neurotoxicity and gliotoxicity

Cytostatic drugs utilize various mechanisms via which they attack cancers. Alkylating agents alkylate electron-rich atoms, form covalent bonds, and react with DNA bases. This reaction prevents cells from replicating (39). Cisplatin and analogues form monofunctional and bifunctional adducts which lead to intrastrand or interstrand DNA cross-links interrupting separation, replication and transcription of the DNA (39). Antimetabolites disturb the biosynthesis and function of nucleic acids and impair formation of new DNA and RNA, which leads to cell cycle arrest. Finally, DNA topoisomerase inhibitors form single- or double-strand breaks in the DNA double helix, which relaxes the torsional stress that occurs when the DNA double helix unwinds. Accumulation of torsionally strained and supercoiled DNA interferes with vital cell processes leading to cytotoxic DNA damage causing cell arrest, apoptosis, or necrosis [38].

There has been considerable preclinical research, which demonstrated that cytostatic drugs can produce cognitive impairment in small animal models (for review, see [39–41]). The pathomechanisms of this toxicity have been explored in vivo and in vitro.

It appears that toxicity induced by cytostatic drugs within the central nervous system utilizes pathways which are also involved in other brain injury syndromes such as hypoxia/ischemia, traumatic injury, and neuroinflammation.

*Oxidative stress* is attributed to disruption of mitochondrial DNA leading to formation of reactive oxygen species (ROS). Production of ROS has been demonstrated for various agents, including cyclophosphamide, cytarabin, doxorubicin, methotrexate, and carboplatin [42–51].

*Disruption of neurogenesis* has been shown to occur within the hippocampus following treatment with carmustine, cyclophosphamide, 5-fluorouracil, or cisplatin [40, 41, 53–58].

#### Excitotoxicity and apoptosis

In many acute and chronic brain injury syndromes, such as hypoxia-ischemia, trauma, status epilepticus, and neurodegeneration in the context of mitochondrial dysfunction [59–62], toxic stimuli operate via two well-characterized mechanisms to cause neuronal death. *Excitotoxicity* is a form of passive neuronal death caused by excessive stimulation of excitatory amino acid (EAA) receptors [60–62]. Three subtypes of EAA receptors, *N*-methyl-d-aspartate, alpha-amino-3-hydroxyl-5-methyl-isoxazol-4-propionic acid, and kainate receptors, are coupled to ion channels and are called ionotropic. Excessive stimulation of ionotropic glutamate receptors causes excitotoxic neuronal death in vitro and in vivo [63]. Active caspase-mediated cell death or *apoptosis* represents a form of slower degeneration that occurs in hypoxic and traumatic brain injury as well as in the context of mitochondrial dysfunction [59, 60, 62, 64]. Caspase-mediated cell death can be triggered by a primary excitotoxic stimulus of low intensity [65].

In the developing brain, active cell death that occurs after hypoxic or traumatic brain injury markedly resembles morphologically physiological apoptosis [59, 66, 67]. Rzeski and colleagues undertook a series of studies aimed to evaluate whether anticancer agents may exert direct neurotoxic effects and also explored whether excitotoxic and caspase-mediated death comprise components of this toxicity. They investigated neurotoxic effects of common cytotoxic drugs in vitro in neuronal and glial cultures and in vivo in the developing rat brain [68]. When neurons and astroglia were exposed to cisplatin, cyclophosphamide, methotrexate, vinblastin, or thiotepa, a concentration-dependent neurotoxic effect was observed. Neurotoxicity was potentiated by nontoxic glutamate concentrations and blocked by ionotropic glutamate receptor antagonists and a pancaspase inhibitor. To investigate neurotoxicity in vivo, Rzeski and colleagues administered to infant rats cisplatin, cyclophosphamide, thiotepa, or ifosfamide and analyzed their brains. All tested compounds produced widespread lesions within cortex, thalamus, hippocampal dentate gyrus, and caudate nucleus in a dose-dependent fashion [68]. Early histological analysis demonstrated dendritic swelling and relative preservation of axonal terminals, which are morphological features indicating excitotoxicity. After longer survival periods, degenerating neurons displayed morphological features consistent with active, caspase-mediated cell death. These results demonstrate that anticancer drugs are potent neurotoxins in vitro and in vivo; they activate excitotoxic mechanisms but also trigger active, caspase-mediated neuronal death. Other investigators have reported similar findings [46, 69, 70].

A direct toxic effect of some cytostatic drugs on oligodendrocytes and their precursors has been described [52, 54, 71] and likely contributes to white matter pathology seen in pediatric cancer survivors.

#### Neuroinflammation

Systemic inflammation with cytokine release, which may occur in cancer patients as a response to mucositis and systemic infections, may facilitate a process of *neuroinflammation*, microglial activation, and suppression of neurogenesis [72–77].

#### Brain perfusion

A reduction by chemotherapy of regional blood flow, possibly due to a reduction in blood vessel density, has also been reported [78–80], including more recently a clinical study in pediatric cancer survivors [81]. Using positron emission tomography/magnetic resonance imaging (PET/MR), these investigators measured significantly lower cerebral blood flow and metabolic activity in key brain areas compared to control subjects.

#### The role of the blood–brain barrier

The brain is protected against potentially harmful medications by the blood–brain barrier, which consist of capillary endothelial unfenestrated cells, linked by tight junctions. Efflux transporters such as P-glycoprotein control and limit invasion of cytotoxic drugs [39]. Moreover, pericytes inhibit the expression of molecules that increase vascular permeability and CNS immune cell infiltration [82]. Multiple studies suggest that the blood–brain barrier is already mature and effective in the fetal brain [83, 84].

To penetrate the blood–brain barrier, drug molecules need to be small (< 500 Da) and lipophilic so they can passively diffuse. Drugs that can use inward transport systems but remain unrecognized by efflux transporters, can also enter the brain [85]. The blood–brain barrier can be disrupted in the proximity to brain tumors and brain metastases, posterior reversible encephalopathy syndrome, following radiation and when brain disruptors are used [86]. In these cases, chemotherapeutic agents can easily penetrate into the central nervous system. Furthermore, in a number of pediatric malignancies, intrathecal chemotherapy is administered which increases the risk for neurologic complications.

### Chemotherapy-induced neuropathology

Some cancer chemotherapeutics have been studied in rodents. The studies have focused on methotrexate, alkylating agents (ifosfamide, cyclophosphamide, cisplatin), and vincristine (see reviews [5, 39]). Neuropathological and neurophysiological correlates of CNS toxicity in infant, young, and adult rodents have included marked increase of apoptosis, decline in neurogenesis, impairment of long-term potentiation (LTP), synaptic remodeling, increased blood–brain barrier permeability, impaired cell division and migration, and increased markers for oxidative stress [5, 39]. Table 1 summarizes the types of CNS toxicity described in rodent models at different ages.

**Table 1:** Neurotoxic effects of chemotherapeutic agents in rodent models [25]

Drug	Form of neurotoxicity	Age	Brain region
Methotrexate *antimetabolite*	Apoptotic and excitotoxic neuronal death, decline in neurogenesis, decrease in myelination, oxidative stress	7 days 3 months 3 months 12 months	Cortex, thalamus, caudate nucleus, hippocampus, corpus callosum, cerebellum, pons, medulla, hypothalamus [40, 41, 50, 68, 87, 88]
Cyclophosphamide Ifosfamide *alkylating*	Apoptotic and excitotoxic neuronal death, decline in neurogenesis, impairment of LTP, cytokine dysregulation, reduced glutathione, and glutathione peroxidase	7 days 8–10 weeks 2 months Adult 7 months 12 months Embryo	Cortex, thalamus, caudate nucleus, hippocampus, corpus callosum, neural tube [58, 68, 87–91]
Cisplatin *alkylating*	Apoptotic and excitotoxic neuronal death, Decreased cell division, altered granule cell migration and Purkinje cell dendrite growth, increased blood–brain barrier permeability, DNA damage due to oxidative stress	7 days 10 days 8 days 1 day Embryo Adult	Cortex, thalamus, caudate nucleus, hippocampus, cerebellar cortex, cerebellar granule neurons, hypothalamus [52, 68, 70, 92–98]
Vincristine *anti-mitotic*	Apoptosis	8 days	Cortex, thalamus, caudate nucleus, hippocampus [68, 70]

In humans, brain pathology can be studied using MRI techniques. Assigning a particular type of toxicity to one medication is very difficult, given the fact that multidrug regimen are used to treat pediatric malignancies. In addition, radiotherapy is often coadministered.

A most recent study by van der Plas and colleagues in survivors of childhood B-cell ALL who received no radiotherapy describes smaller volumes of both grey and white matter structures, indicating that there has been cell loss in these areas and/or their development was compromised [99]. Nevertheless, there are distinct forms of brain toxicity that have been associated with certain chemotherapeutic drugs.

*Leukoencephalopathy* is a known complication of chemotherapy, in children and adults, in particular for regimens that include methotrexate, BCNU, melphalan, fludarabine, cytarabine, 5-fluorouracil, levamisole, and cisplatin [100–104]. In many cases, a mild and reversible form of injury occurs. When methotrexate is combined with radiation therapy, the degree of white matter injury increases dramatically and, in such cases, leukoencephalopathy may be irreversible. Determining the relative contributions of each treatment modality to brain injury is practically impossible under such circumstances [101, 102, 105–109].

However, methotrexate has been shown to cause the same type of toxic leukoencephalopathy in the absence of radiation, in cases with intrathecal or intraventricular administration [110–113]. The greatest injury is seen surrounding a leaky or misplaced ventriculostomy tube used to administer methotrexate via an Ommaya reservoir [100, 109, 114–116]. Risk factors for toxicity are also not well understood, but appear to relate to dosages of methotrexate and radiation, modes of administration, types of diluent, preexisting folate deficiency, and idiosyncratic predispositions [86, 101].

An early asymptomatic form of leukoencephalopathy has been reported in children with medulloblastoma and supratentorial primary neuroectodermal tumors receiving combination chemotherapy, with or without concomitant radiation [117–119]. In most cases, these lesions were transient and reversible, but there was an increased risk of subsequent neurocognitive deficits. An acute and transient form of encephalopathy has been reported in children receiving high-dose methotrexate for acute lymphoblastic leukemia or osteosarcoma [120]. It has been postulated that under such circumstances a disruption of the blood–brain barrier occurs. In biopsies of such lesions, myelin pallor, vacuolation, axonal spheroids, modest macrophage infiltrates, and gliosis have been reported [86].

*Disseminated necrotizing leukoencephalopathy (DNL)* presents with a miliary distribution of lesions, ranging from small rounded foci to large confluent zones of non-inflammatory demyelination or white matter necrosis [86, 108, 121, 122]. This disease was first described in children with metastatic meningeal acute lymphoblastic lymphoma (ALL) treated with high-dose methotrexate-based chemotherapy and whole brain irradiation [108, 123]. In adults, DNL has been described in patients with other tumor types, primary CNS and systemic lymphomas, carcinomas, sarcomas, and primitive/embryonal neoplasms [100, 106, 107, 111, 124–127], and in high-grade gliomas treated with intra-arterial BCNU, both with and without irradiation [128–131].

Clinical presentation of DNL is that of a rapidly progressive subcortical dementia. Symptoms present after completion of therapy to many months later and may progress to dementia, seizures, coma, and death within months [86].

#### Reversible posterior leukoencephalopathy syndrome

Reversible posterior leukoencephalopathy syndrome (RPLES) or posterior reversible encephalopathy syndrome (PRES) presents with acute cortical blindness, headache, mental status changes, and sometimes seizures [132]. Malignant hypertension and T2-weighted/FLAIR MRI signal abnormalities in the occipital and posterior temporo-parietal regions are the hallmarks of this disease entity. Lesions may affect grey matter [133–140]. High-dose corticosteroids and various single or combination chemotherapeutic regimens, including cisplatin, cytarabine, cyclophosphamide, and methotrexate, have been identified as triggers.

Symptoms may develop at initiation of therapy or may be delayed for days to weeks. The mechanism of toxicity is poorly understood, although radiologic studies suggest vasogenic edema as the main pathology. Proposed mechanisms have included endothelial damage with blood–brain barrier disruption, transient episodes of hypertension overloading the autoregulatory capabilities of the posterior circulation, and electrolyte imbalances, such as hypomagnesemia [133–140]. In rarely obtained biopsies, vasogenic edema without vascular damage or infarct was detected [137]. In some patients, permanent deficits were encountered suggesting that ischemic damage is possible in severe cases or delays in making the diagnosis and instituting blood pressure control.

### Future challenges

There is increasing knowledge from preclinical studies about the effects of cancer chemotherapeutic agents on the mammalian brain, but little information is available on how such findings from various animal models translate and apply to the human pediatric brain.

Advanced neuroimaging studies in cancer patients have started to shed light on the structural and functional impact of chemotherapy on the pediatric and adult brain. There are few studies focusing on dynamic changes in brain morphology and function during chemotherapy in children, and there is a dearth of longitudinal prospective clinical studies that examine step by step their progression and potential reversibility. This is the type of studies needed to help explore what acute effects cancer and cancer treatment exert on the developing brain, how early they occur, what the earliest indicators and mechanisms are, and whether treatments can be developed to counteract and/or prevent them.

Better understanding of pathomechanisms and identification of biomarkers which can trace neurotoxicity risk in individual patients will allow for timely modifications of treatment to minimize toxicity.

## Abbreviations

5-FU: 5-Fluorouracil
ALL: Acute lymphoblastic leukemia
BBB: Blood–brain barrier
CNS: Central nervous system
CSF: Cerebrospinal fluid
DNA: Deoxyribonucleic acid
DNL: Disseminated necrotizing leukoencephalopathy
DTI: Diffusion tensor imaging
EAA: Excitatory amino acid
FA: Fractional anisotropy
FIRST: FMRIB’s Integrated Registration and Segmentation Tool
GM: Grey matter
IQ: Intelligence quotient
MR: Magnetic resonance
PET: Positron emission tomography
PRES: Posterior reversible encephalopathy syndrome
ROS: Reactive oxygen species
RPLES: Reversible posterior leukoencephalopathy syndrome
VBM: Voxel based morphometry
WM: White matter

## References

[1] Henderson TO, Friedman DL, Meadows AT (2010). Childhood cancer survivors: transition to adult-focused risk-based care. Pediatrics.

[2] Kadan-Lottick NS, Zeltzer LK, Liu Q (2010). Neurocognitive functioning in adult survivors of childhood noncentral nervous system cancers. J Natl Cancer Inst.

[3] Oeffinger KC, Nathan PC, Kremer LC (2010). Challenges after curative treatment for childhood cancer and long-term follow up of survivors. Hematol Oncol Clin North Am.

[4] Zeltzer LK, Recklitis C, Buchbinder D (2009). Psychological status in childhood cancer survivors: a report from the Childhood Cancer Survivor Study. J Clin Oncol.

[5] Sleurs C, Lemiere J, Vercruysse T (2017). Intellectual development of childhood ALL patients: a multicenter longitudinal study. Psycho-Oncology.

[6] Duffner PK, Armstrong FD, Chen L (2014). Neurocognitive and neuroradiologic central nervous system late effects in children treated on Pediatric Oncology Group (POG) P9605 (standard risk) and P9201 (lesser risk) acute lymphoblastic leukemia protocols (ACCL0131): a methotrexate consequence? A report from the Children’s Oncology Group. J Pediatr Hematol Oncol.

[7] Anderson FS, Kunin-Batson AS (2009). Neurocognitive late effects of chemotherapy in children: the past 10 years of research on brain structure and function. Pediatr Blood Cancer.

[8] Ashford J, Schoffstall C, Reddick WE (2010). Attention and working memory abilities in children treated for acute lymphoblastic leukemia. Cancer.

[9] Buizer AI, de Sonneville LM, Veerman AJ (2009). Effects of chemotherapy on neurocognitive function in children with acute lymphoblastic leukemia: a critical review of the literature. Pediatr Blood Cancer.

[10] Lofstad GE, Reinfjell T, Hestad K (2009). Cognitive outcome in children and adolescents treated for acute lymphoblastic leukaemia with chemotherapy only. Acta Paediatr.

[11] Moleski M (2000). Neuropsychological, neuroanatomical, and neurophysiological consequences of CNS chemotherapy for acute lymphoblastic leukemia. Arch Clin Neuropsychol.

[12] von der Weid N, Mosimann I, Hirt A (2003). Intellectual outcome in children and adolescents with acute lymphoblastic leukaemia treated with chemotherapy alone: age- and sex-related differences. Eur J Cancer.

[13] Jansen NC, Kingma A, Schuitema A (2008). Neuropsychological outcome in chemotherapy-only-treated children with acute lymphoblastic leukemia. J Clin Oncol.

[14] Bhojwani D, Sabin ND, Pei D (2014). Methotrexate-induced neurotoxicity and leukoencephalopathy in childhood acute lymphoblastic leukemia. J Clin Oncol.

[15] Carey ME, Haut MW, Reminger SL (2008). Reduced frontal white matter volume in long-term childhood leukemia survivors: a voxel-based morphometry study. AJNR Am J Neuroradiol.

[16] Genschaft M, Huebner T, Plessow F (2013). Impact of chemotherapy for childhood leukemia on brain morphology and function. PLOS One.

[17] Asato R, Akiyama Y, Ito M (1992). Nuclear magnetic resonance abnormalities of the cerebral white matter in children with acute lymphoblastic leukemia and malignant lymphoma during and after central nervous system prophylactic treatment with intrathecal methotrexate. Cancer.

[18] Dellani PR, Eder S, Gawehn J (2008). Late structural alterations of cerebral white matter in long-term survivors of childhood leukemia. J Magn Reson Imaging.

[19] Reddick WE, Glass JO, Johnson DP, Laningham FH, Pui C-H (2009). Voxel-based analysis of T2 hyperintensities in white matter during treatment of childhood leukemia. Am J Neuroradiol.

[20] Deprez S, Amant F, Smeets A (2012). Longitudinal assessment of chemotherapy-induced changes in cerebral white matter and its correlation with impaired cognitive functioning. J Clin Oncol.

[21] Khong P-L, Leung LHT, Fung ASM (2006). White matter anisotropy in post-treatment childhood cancer survivors: preliminary evidence of association with neurocognitive function. J Clin Oncol.

[22] Edelmann MN, Krull KR, Liu W (2014). Diffusion tenson imaging and neurocognition in survivors of childhood acute lymphoblastic leukaemia. Brain.

[23] ElAlfy M, Ragab I, Azab I, Amin S, Abdel-Maguid M (2014). Neurocognitive outcome and white matter anisotropy in childhood acute lymphoblastic leukemia survivors treated with different protocols. Pediatr Hematol Oncol.

[24] Kesler SR, Gugel M, Huston-Warren E, Watson C (2016). Atypical structural connectome organization and cognitive impairment in young survivors of acute lymphoblastic leukemia. Brain Connect.

[25] Sleurs C, Lemiere J, Christiaens D et al (2018) Advanced MR diffusion imaging and chemotherapy-related changes in cerebral white matter microstructure of survivors of childhood bone and soft tissue sarcoma. Hum Brain Mapp:1–13 10.1002/hbm.24082 PMC6866599 29675944

[26] McDonald BC, Conroy SK, Ahles TA, West JD, Saykin AJ (2010). Gray matter reduction associated with systemic chemotherapy for breast cancer: a prospective MRI study. Breast Canc Res Treat.

[27] McDonald BC, Conroy SK, Smith DJ, West JD, Saykin AJ (2013). Frontal gray matter reduction after breast cancer chemotherapy and association with executive symptoms: a replication and extension study. Brain Behav Immun.

[28] Tamnes CT, Zeller B, Amlien IK (2015). Cortical surface area and thickness in adult survivors of pediatric acute lymphoblastic leukemia. Pediatr Blood Cancer.

[29] Oesterlundh G, Kjellmer I, Lannering B (2008). Neurochemical markers of brain damage in cerebrospinal fluid during induction treatment of acute lymphoblastic leukemia in children. Pediatr Blood Cancer.

[30] Quinn CT, Griener JC, Bottiglieri T (1997). Elevation of homocysteine and excitatory amino acid neurotransmitters in the CSF of children who receive methotrexate for the treatment of cancer. J Clin Oncol.

[31] Viacha V, Eliopoulou M, Haidas S, Beratis NG (2004). Correlation of cerebrospinal fluid betal-glucuronidase activity with plasma methotrexate concentrations in leukemic children receiving high-dose methotrexate. Pediatr Blood Cancer.

[32] Chiaretti A, Ruggiero A, Coccia P (2011). Expression of liquoral neuroprotection markers in children with acute lymphoblastic leukemia. Leukemia Res.

[33] Van Gool SW, De Meyer G, van de Voorde A, Vanmechelen E, Vanderstichele H (2004). Neurotoxicity marker profiles in the CSF are not age-dependent but show variation in children treated for acute lymphoblastic leukemia. Neurotoxicology.

[34] Krawczuk-Rybak M, Grabowska A, Protal PT, Muszynska-Roslan K, Braszko J (2012). Intellectual functioning of childhood leukemia survivors – relation to Tau protein – a marker of white matter injury. Adv Med Sci.

[35] Caron JE, Krull KR, Hockenberry M (2009). Oxidative stress and executive function in children receiving chemotherapy for acute lymphoblastic leukemia. Pediatr Blood Cancer.

[36] Taylor OA, Hockenberry MJ, McCarthy K (2015). Evaluation of biomarkers of oxidative stress and apoptosis in patients with severe methotrexate neurotoxicity: a case series. J Pediatr Oncol Nurs.

[37] Cole PD, Beckwith KA, Vijayanathan V (2009). Folate homeostasis in cerebrospinal fluid during therapy for acute lymphoblastic leukemia. Pediatr Neurol.

[38] DeVita V, Hellman S, Rosenberg S (2005). Cancer: principles&practice of oncology.

[39] Seigers R, Fardell JE (2011). Neurobiological basis of chemotherapy-induced cognitive impairment: a review of rodent research. Neurosci Biobehav Rev.

[40] Seigers R, Schagen SB, Beerling W (2008). Long-lasting suppression of hippocampal cell proliferation and impaired cognitive performance by methotrexate in the rat. Behav Brain Res.

[41] Seigers R, Schagen SB, Coppens CM (2009). Methotrexate decreases hippocampal cell proliferation and induces memory deficits in rats. Behav Brain Res.

[42] Geller HM, Cheng KY, Goldsmith NK (2001). Oxidative stress mediates neuronal DNA damage and apoptosis in response to cytosine arabinoside. J Neurochem.

[43] Husain K, Whitworth C, Hazelrigg S, Rybak L (2003). Carboplatin-induced oxidative injury in rat inferior colliculus. Int J Toxicol.

[44] Husain K, Whitworth C, Somani SM, Rybak LP (2001). Carboplatin-induced oxidative stress in rat cochlea. Hear Res.

[45] Oboh G, Ogunruku OO (2010). Cyclophosphamide-induced oxidative stress in brain: protective effect of hot short pepper (Capsicum frutescens L. var. abbreviatum). Exp Toxicol Pathol.

[46] Koros C, Kitraki E (2009). Neurofilament isoform alterations in the rat cerebellum following cytosine arabinoside administration. Toxicol Lett.

[47] Joshi G, Sultana R, Tangpong J (2005). Free radical mediated oxidative stress and toxic side effects in brain induced by the anti cancer drug adriamycin: insight into chemobrain. Free Radic Res.

[48] Montilla P, Tunez I, Munoz MC, Soria JV, Lopez A (1997). Antioxidative effect of melatonin in rat brain oxidative stress induced by Adriamycin. Rev Esp Fisiol.

[49] Öz E, Ilhan MN (2006). Effects of melatonin in reducing the toxic effects of doxorubicin. Mol Cell Biochem.

[50] Rajamani R, Muthuvel A, Senthilvelan M, Sheeladevi R (2006). Oxidative stress induced by methotrexate alone and in the presence of methanol in discrete regions of the rodent brain, retina and optic nerve. Toxicol Lett.

[51] Uzar E, Koyuncuoglu HR, Uz E (2006). The activities of antioxidant enzymes and the level of malondialdehyde in cerebellum of rats subjected to methotrexate: protective effect of caffeic acid phenethyl ester. Mol Cell Biochem.

[52] Dietrich J, Han R, Yang Y, Mayer-Proschel M, Noble M (2006). CNS progenitor cells and oligodendrocytes are targets of chemotherapeutic agents in vitro and in vivo. J Biol.

[53] Dietrich J, Prust M, Kaiser J (2015). Chemotherapy, cognitive impairment and hippocampal toxicity. Neuroscience.

[54] Han R, Yang YM, Dietrich J (2008). Systemic 5-fluorouracil treatment causes a syndrome of delayed myelin destruction in the central nervous system. J Biol.

[55] Mignone RG, Weber ET (2006). Potent inhibition of cell proliferation in the hippocampal dentate gyrus of mice by the chemotherapeutic drug thioTEPA. Brain Res.

[56] Mondie CM, Vandergrift KA, Wilson CL, Gulinello ME, Weber ET (2010). The chemotherapy agent, thioTEPA, yields long-term impairment of hippocampal cell proliferation and memory deficits but not depression-related behaviors in mice. Behav Brain Res.

[57] Mustafa S, Walker A, Bennett G, Wigmore PM (2008). 5-Fluorouracil chemotherapy affects spatial working memory and newborn neurons in the adult rat hippocampus. Eur J Neurosci.

[58] Yang M, Kim JS, Song MS (2010). Cyclophosphamide impairs hippocampus-dependent learning and memory in adult mice: possible involvement of hippocampal neurogenesis in chemotherapy-induced memory deficits. Neurobiol Learn Mem.

[59] Bittigau P, Sifringer M, Pohl D (1999). Apoptotic neurodegeneration following trauma is markedly enhanced in the immature brain. Ann Neurol.

[60] Bossy-Wetzel E, Barsoum MJ, Godzik A (2003). Mitochondrial function in apoptosis, neurodegeneration and aging. Curr Opin Cell Biol.

[61] Lipton SA, Rosenberg PA (1994). Excitatory amino acids as a final common pathway for neurologic disorders. N Engl J Med.

[62] Murphy AN, Fiskum G, Beal MF (1999). Mitochondria in neurodegeneration: bioenergetic function in cell life and death. J Cereb Blood Flow Metab.

[63] Rothman SM, Olney JW (1995). Excitotoxicity and the NMDA receptor—still lethal after eight years. Trends Neurosci.

[64] Lee JM, Zipfel GJ, Choi DW (1999). The changing landscape of ischaemic brain injury mechanisms. Nature.

[65] Bonfoco E, Krainc D, Ankarcrona M (1995). Apoptosis and necrosis: two distinct events induced, respectively, by mild and intense insults with N-methyl-D-aspartate or nitric oxide/superoxide in cortical cell cultures. Proc Natl Acad Sci U S A.

[66] Northington FJ, Ferriero DM, Graham EM (2001). Early neurodegeneration after hypoxia-ischemia in neonatal rat is necrosis while delayed neuronal death is apoptosis. Neurobiol Dis.

[67] Pohl D, Bittigau P, Ishimaru MJ (1999). NMDA antagonists and apoptotic cell death triggered by head trauma in developing rat brain. Proc Natl Acad Sci U S A.

[68] Rzeski W, Pruskil S, Macke A (2004). Anticancer agents are potent neurotoxins in vitro and in vivo. Ann Neurol.

[69] Courtney MJ, Coffey ET (1999). The mechanism of Ara-C-induced apoptosis of differentiating cerebellar granule neurons. Eur J Neurosci.

[70] Wick A, Wick W, Hirrlinger J (2004). Chemotherapy-induced cell death in primary cerebellar granule neurons but not in astrocytes: in vitro paradigm of differential neurotoxicity. J Neurochem.

[71] Gregorios JB, Gregorios AB, Mora J (1989). Morphologic alterations in rat brain following systemic and intraventricular methotrexate injection: light and electron microscopic studies. J Neuropathol Exp Neurol.

[72] Das S, Basu A (2008). Inflammation: a new candidate in modulating adult neurogenesis. J Neurosci Res.

[73] de Koning BA, van Dieren JM, Lindenbergh-Kortleve DJ (2006). Contributions of mucosal immune cells to methotrexate-induced mucositis. Int Immunol.

[74] De Visser KE, Eichten A, Coussens LM (2006). Paradoxical roles of the immune system during cancer development. Nat Rev Cancer.

[75] Ekdahl CT, Claasen JH, Bonde S, Kokaia Z, Lindvall O (2003). Inflammation is detrimental for neurogenesis in adult brain. Proc Natl Acad Sci U S A.

[76] Seruga B, Zhang H, Bernstein LJ, Tannock IF (2008). Cytokines and their relationship to the symptoms and outcome of cancer. Nat Rev Cancer.

[77] Wilson CJ, Finch CE, Cohen HJ (2002). Cytokines and cognition—the case for a head-to-toe inflammatory paradigm. J Am Geriatr Soc.

[78] de Vos FY, Willemse PH, De Vries EG, Gietema JA (2004). Endothelial cell effects of cytotoxics: balance between desired and unwanted effects. Cancer Treat Rev.

[79] Mizusawa S, Kondoh Y, Murakami M (1988). Effect of methotrexate on local cerebral blood flow in conscious rats. Jpn J Pharmacol.

[80] Seigers R, Timmermans J, van der Horn HJ (2010). Methotrexate reduces hippocampal blood vessel density and activates microglia in rats but does not elevate central cytokine release. Behav Brain Res.

[81] Theruvath AJ, Ilivitzki A, Muehe A (2017). A PET/MRI imaging approach for the integrated assessment of chemotherapy-induced brain, heart, and bone injuries in pediatric cancer survivors: a pilot study. Radiology.

[82] Daneman R, Zhou L, Kebede AA, Barres BA (2010). Pericytes are required for blood-brain barrier integrity during embryogenesis. Nature.

[83] Saunders NR, Knott GW, Dziegielewska KM (2000). Barriers in the immature brain. Cell Mol Neurobiol.

[84] Virgintino D, Errede M, Girolamo F (2008). Fetal blood-brain barrier P-glycoprotein contributes to brain protection during human development. J Neuropathol Exp Neurol.

[85] de Vries NA, Beijnen JH, Boogerd W, van Tellingen O (2006). Blood–brain barrier and chemotherapeutic treatment of brain tumors. Expert Rev Neurother.

[86] Perry A, Schmidt RE (2006). Cancer therapy-associated CNS neuropathology: an update and review of the literature. Acta Neuropathol.

[87] Briones TL, Woods J (2011). Chemotherapy-induced cognitive impairment is associated with decreases in cell proliferation and histone modifications. BMC Neurosci.

[88] Briones TL, Woods J (2013). Dysregulation in myelination mediated by persistent neuroinflammation: possible mechanisms in chemotherapy-related cognitive impairment. Brain Behav Immun.

[89] Lyons L, Elbeltagy M, Bennett G, Wigmore P (2011). The effects of cyclophosphamide on hippocampal cell proliferation and spatial working memory in rat. PLoS One.

[90] Lee GD, Longo DI, Wang Y (2006). Transient improvement in cognitive function and synaptic plasticity in rats following cancer chemotherapy. Clin Cancer Res.

[91] Xiao R, Yu HL, Zhao HF (2007). Developmental neurotoxicity role of cyclophosphamide onpost-neural tube closure of rodents in vitro and in vivo. Int J Dev Neurosci.

[92] Avella D, Pisu MB, Roda E, Gravati M, Bernocchi G (2006). Reorganization of the rat cerebellar cortex during postnatal development following cisplatin treatment. Exp Neurol.

[93] Andres AL, Gong C, Di K, Bota DA (2014). Low-doses of cisplatin injure hippocampal synapses: a mechanism for ‘chemo’ brain?. Exp Neurol.

[94] Cerri S, Piccolini VM, Santin G (2011). The developmental neurotoxicity study of platinum compounds: effects of cisplatin versus a novel Pt(II) complex on rat cerebellum. Neurotoxicol Teratol.

[95] Gopal KV, Wu C, Shrestha B (2012). D-Methionine protects against cisplatin-induced neurotoxicity in cortical networks. Neurotoxicol Teratol.

[96] Sugimoto S, Yamamoto YL, Nagahiro S, Diksic M (1995). Permeability change and brain tissue damage after intracarotid administration of cisplatin studied by double-tracer autoradiography in rats. J Neuro-Oncol.

[97] Piccolini VM, Cerri S, Romanelli E, Bernocchi G (2012). Interactions of neurotransmitter systems duringpostnatal development of the rat hippocampal formation: effects of cisplatin. Exp Neurol.

[98] Turan MI, Cayir A, Cetin N (2014). An investigation of the effect of thiamine pyrophosphateon cisplatin-induced oxidative stress and DNA damage in rat brain tissue compared with thiamine: thiamine and thiamine pyrophosphate effects on cisplatin neurotoxicity. Hum Exp Toxicol.

[99] van der Plas E, Schachar RJ, Hitzler J (2016). Brain structure, working memory and response inhibition in childhood leukemia survivors. Brain Behav.

[100] Cossaart N, SantaCruz KS, Preston D, Johnson P, Skikne BS (2003). Fatal chemotherapy-induced encephalopathy following high-dose therapy for metastatic breast cancer: a case report and review of the literature. Bone Marrow Transplant.

[101] Cruz-Sanchez FF, Artigas J, Cervos-Navarro J, Rossi ML, Ferszt R (1991). Brain lesions following combined treatment with methotrexate and craniospinal irradiation. J Neuro-Oncol.

[102] Fassas AB, Gattani AM, Morgello S (1994). Cerebral demyelination with 5-fluorouracil and levamisole. Cancer Investig.

[103] Liu HM, Maurer HS, Vongsvivut S, Conway JJ (1978). Methotrexate encephalopathy. A neuropathologic study. Hum Pathol.

[104] Moore-Maxwell CA, Datto MB, Hulette CM (2004). Chemotherapy-induced toxic leukoencephalopathy causes a wide range of symptoms: a series of four autopsies. Mod Pathol.

[105] Antunes NL, Souweidane MM, Lis E, Rosenblum MK, Steinherz PG (2002). Methotrexate leukoencephalopathy presenting as Kluver–Bucy syndrome and uncinate seizures. Pediatr Neurol.

[106] DeAngelis LM, Seiferheld W, Schold SC, Fisher B, Schultz CJ (2002). Combination chemotherapy and radiotherapy for primary central nervous system lymphoma: radiation therapy oncology group study 93–10. J Clin Oncol.

[107] Lai R, Abrey LE, Rosenblum MK, DeAngelis LM (2004). Treatment-induced leukoencephalopathy in primary CNS lymphoma: a clinical and autopsy study. Neurology.

[108] Rubinstein LJ, Herman MM, Long TF, Wilbur JR (1975). Disseminated necrotizing leukoencephalopathy: a complication of treated central nervous system leukemia and lymphoma. Cancer.

[109] Stone JA, Castillo M, Mukherji SK (1999). Leukoencephalopathy complicating an Ommaya reservoir and chemotherapy. Neuroradiology.

[110] Abelson HT (1978). Methotrexate and central nervous system toxicity. Cancer Treat Rep.

[111] Allen JC, Rosen G, Mehta BM, Horten B (1980). Leukoencephalopathy following high-dose iv methotrexate chemotherapy with leucovorin rescue. Cancer Treat Rep.

[112] Omuro A. M.P., DeAngelis L. M., Yahalom J., Abrey L. E. (2005). Chemoradiotherapy for primary CNS lymphoma: An intent-to-treat analysis with complete follow-up. Neurology.

[113] Lovblad K, Kelkar P, Ozdoba C (1998). Pure methotrexate encephalopathy presenting with seizures: CT and MRI features. Pediatr Radiol.

[114] de Waal R, Algra PR, Heimans JJ, Wolbers JG, Scheltens P (1993). Methotrexate induced brain necrosis and severe leukoencephalopathy due to disconnection of an Ommaya device. J Neuro-Oncol.

[115] Packer RJ, Zimmerman RA, Rosenstock J (1981). Focal encephalopathy following methotrexate therapy. Administration via a misplaced intraventricular catheter. Arch Neurol.

[116] Colamaria V, Caraballo R, Borgna-Pignatti C (1990). Transient focal leukoencephalopathy following intraventricular methotrexate and cytarabine. A complication of the Ommaya reservoir: case report and review of the literature. Childs Nerv Syst.

[117] Fouladi M, Chintagumpala M, Laningham FH (2004). White matter lesions detected by magnetic resonance imaging after radiotherapy and high-dose chemotherapy in children with medulloblastoma or primitive neuroectodermal tumor. J Clin Oncol.

[118] Rutkowski S, Bode U, Deinlein F (2005). Treatment of early childhood medulloblastoma by postoperative chemotherapy alone. N Engl J Med.

[119] Fouladi Maryam, Langston James, Mulhern Raymond, Jones Dana, Xiong Xiaoping, Yang Jianping, Thompson Stephen, Walter Andrew, Heideman Richard, Kun Larry, Gajjar Amar (2000). Silent Lacunar Lesions Detected by Magnetic Resonance Imaging of Children With Brain Tumors: A Late Sequela of Therapy. Journal of Clinical Oncology.

[120] Rubnitz JE, Relling MV, Harrison PL (1998). Transient encephalopathy following high-dose methotrexate treatment in childhood acute lymphoblastic leukemia. Leukemia.

[121] Price RA, Jamieson PA (1975). The central nervous system in childhood leukemia. II. Subacute leukoencephalopathy. Cancer.

[122] Smith B (1975). Brain damage after intrathecal methotrexate. J Neurol Neurosurg Psychiatry.

[123] Rubinstein JL, Herman MM, Long TF, Wilbur JR (1975). Leukoencephalopathy following combined therapy of central nervous system leukemia and lymphoma. Acta Neuropathol Suppl (Berl) Suppl.

[124] Atlas SW, Grossman RI, Packer RJ (1987). Magnetic resonance imaging diagnosis of disseminated necrotizing leukoencephalopathy. J Comput Tomogr.

[125] Batara JF, Grossman SA (2003). Primary central nervous system lymphomas. Curr Opin Neurol.

[126] Sindwahni G, Arora M, Thakker VD, Jain A (2017) MRI in chemotherapy induced leukoencephalopathy: report of two cases and radiologist's perspective. J Clin Diagn Res TD08-TD09. 10.7860/JCDR/2017/29164.10248. 10.7860/JCDR/2017/29164.10248 PMC5583911 28893007

[127] Omuro AM, Ben-Porat LS, Panageas KS (2005). Delayed neurotoxicity in primary central nervous system lymphoma. Arch Neurol.

[128] Bashir R, Hochberg FH, Linggood RM, Hottleman K (1988). Pre-irradiation internal carotid artery BCNU in treatment of glioblastoma multiforme. J Neurosurg.

[129] Kleinschmidt-DeMasters BK (1986). Intracarotid BCNU leukoencephalopathy. Cancer.

[130] Kleinschmidt-DeMasters BK, Geier JM (1989). Pathology of high-dose intra-arterial BCNU. Surg Neurol.

[131] Rosenblum MK, Delattre JY, Walker RW, Shapiro WR (1989). Fatal necrotizing encephalopathy complicating treatment of malignant gliomas with intra-arterial BCNU and irradiation: a pathological study. J Neuro-Oncol.

[132] Hinchey J, Chaves C, Appignani B (1996). A reversible posterior leukoencephalopathy syndrome. N Engl J Med.

[133] Kahana A, Rowley HA, Weinstein JM (2005). Cortical blindness: clinical and radiologic findings in reversible posterior leukoencephalopathy syndrome: case report and review of the literature. Ophthalmology.

[134] Pavlakis SG, Frank Y, Chusid R (1999). Hypertensive encephalopathy, reversible occipitoparietal encephalopathy, or reversible posterior leukoencephalopathy: three names for an old syndrome. J Child Neurol.

[135] Rangi PS, Partridge WJ, Newlands ES, Waldman AD (2005). Posterior reversible encephalopathy syndrome: a possible late interaction between cytotoxic agents and general anaesthesia. Neuroradiology.

[136] Sanchez-Carpintero R, Narbona J, Lopez de Mesa R, Arbizu J, Sierrasesumaga L (2001). Transient posterior encephalopathy induced by chemotherapy in children. Pediatr Neurol.

[137] Schiff D, Lopes MB (2005). Neuropathological correlates of reversible posterior leukoencephalopathy. Neurocrit Care.

[138] Shin RK, Stern JW, Janss AJ, Hunter JV, Liu GT (2001). Reversible posterior leukoencephalopathy during the treatment of acute lymphoblastic leukemia. Neurology.

[139] Stott VL, Hurrell MA, Anderson TJ (2005). Reversible posterior leukoencephalopathy syndrome: a misnomer reviewed. Intern Med J.

[140] Tam CS, Galanos J, Seymour JF (2004). Reversible posterior leukoencephalopathy syndrome complicating cytotoxic chemotherapy for hematologic malignancies. Am J Hematol.

